# Temporal patterns of motor and nonmotor symptom emergence in Parkinson’s disease: a cluster analysis

**DOI:** 10.1016/j.prdoa.2026.100423

**Published:** 2026-01-06

**Authors:** Yoko Sugimura, Toru Baba, Tomoko Totsune, Hideki Oizumi, Takafumi Hasegawa, Kyoko Suzuki, Atsushi Takeda

**Affiliations:** a Department of Neurology, National Hospital Organization Sendai Nishitaga Hospital, Sendai, Japan; b Department of Cognitive & Motor Aging, Tohoku University Graduate School of Medicine, Sendai, Japan; c Department of Behavioral Neurology and Cognitive Neuroscience, Tohoku University Graduate School of Medicine, Sendai, Japan

**Keywords:** Parkinson’s disease, Nonmotor symptom, Phenotype, REM sleep behavior disorders, Disease progression

## Abstract

**Background:**

Parkinson’s disease is a heterogeneous disorder characterized by a broad spectrum of motor and nonmotor symptoms. Recent studies suggest that the order of motor and nonmotor symptom development may indicate different subtypes of Parkinson’s disease. However, the temporal occurrence patterns of these symptoms remain unclear.

**Objectives:**

This study aims to identify progression patterns of nonmotor symptoms and assess their relationships with the clinical characteristics of Parkinson’s disease.

**Methods:**

We evaluated the prevalence and onset time of motor and nonmotor symptoms in 78 non-demented patients with Parkinson’s disease with a disease duration of less than 10 years, all of whom completed questionnaires regarding the presence of these symptoms and the timing of their appearance. We performed a hierarchical cluster analysis based on onset age and the relative onset times of motor and nonmotor symptoms. We then compared clinical characteristics, and cortical morphological changes among the resulting subgroups. Cortical morphology was analyzed in a subset of patients with available brain imaging and was treated as a sub-analysis.

**Results:**

We identified three core clusters that exhibited different temporal patterns of symptom emergence in Parkinson’s disease. Cluster 1 was an older-onset group with early sleep problems, poor cognitive function. Cluster 2 was a younger-onset group with typical prodromal nonmotor symptom, mostly constipation. Cluster 3 was another older-onset group with poor cognitive function and many nonmotor symptoms present after the onset of motor symptoms. Structural brain imaging was available for most participants (58/78), and a subset of Cluster 1 showed more pronounced cortical atrophy.

**Conclusions:**

Temporal patterns of nonmotor symptom emergence are heterogeneous and are highly influenced by onset age. Older-onset patients showed a higher burden of nonmotor symptoms and worse cognitive function, with symptom emergence occurring either before or after motor symptom onset. Characterizing these symptom-emergence patterns may help refine the clinical classification of Parkinson’s disease and support more individualized evaluation and management.

## Highlights

• Cluster analysis revealed three core temporal patterns of NMS emergence in PD.
• The timing of NMS onset, including prodromal symptoms, showed marked heterogeneity.
• Older-onset patients had many NMS and worse cognition regardless of motor onset.
• Recognizing NMS-emergence patterns may aid PD diagnosis, subtyping and management.

## 1 Introduction

Parkinson’s disease (PD) is the second most common neurodegenerative disorder. It is characterized by a broad spectrum of motor and nonmotor symptoms (NMS) [1,2]. Some NMS, including hyposmia, rapid eye movement behavior disorder (RBD), depression, and constipation, often precede the onset of motor symptoms in PD. These are considered prodromal symptoms. Other NMS, including orthostatic hypotension, cognitive impairment, and psychiatric symptoms, typically emerge after the development of motor symptoms and are regarded as late-stage NMS. It is widely accepted that NMS are a hallmark of PD from the prodromal to the late stages, and the presence of common NMS is among the current diagnostic criteria [3]. Furthermore, several clinical features, such as older age, axial motor symptoms, and specific NMS, including RBD, anosmia, mild cognitive impairment, and orthostatic hypotension, have been shown to be associated with a more severe clinical course of PD [4]. Therefore, an accurate understanding of PD-related NMS is essential to PD diagnosis and treatment.

However, emerging evidence suggests that traditional distinction between prodromal and late-stage NMS may be oversimplified, and that the timing and sequence of specific NMS could instead be linked to prognosis in PD [5]. It has recently been suggested that the progression from typical prodromal NMS to late-stage NMS, previously assumed to be the standard course of PD, may in fact occur only in a small subset of patients [6]. Furthermore, a recent study found that the order of appearance of certain NMS is related to the risk of dementia in PD [7]. It has recently been proposed that RBD preceding motor symptom onset suggests gut-to-brain pathological progression and a more severe clinical course; whereas RBD after the development of motor symptoms indicates brain-to-gut pathological progression and a milder clinical course [8]. However, a single study has reported the opposite finding that RBD appearing after motor symptoms may be linked to a worse prognosis. Given these conflicting findings, clarifying the patterns of symptom combination and the order of appearance of motor symptoms and NMS is expected to be useful for classifying subtypes, predicting prognosis, and estimating likely pathological progression in PD [9].

To clarify the temporal patterns of motor and nonmotor occurence in PD, we conducted a cluster analysis incorporating the order and combinations of these symptoms, together with age at onset, which is known to strongly influence the emergence of NMS [10]. We further conducted a sub-analysis of structural brain MRI using voxel-based morphometry (VBM) to examine whether symptom-emergence–defined clusters are associated with distinct patterns of cortical atrophy.

## 2 Methods

### 2.1 Subjective and clinical examinations

We investigated a total of 121 patients with PD admitted to our hospital between July 2021 and March 2023, all of whom had an MMSE score of 22 or higher. Of these, 6 patients were admitted at the time of initial PD diagnosis (de novo), whereas the remaining patients were hospitalized for medication adjustment and/or rehabilitation. Accordingly, their clinical status was considered relatively stable during the evaluation period. Board-certified neurologists diagnosed PD according to the clinical diagnostic criteria for PD of the Movement Disorder Society (MDS) [3]. Disease onset was defined as the time at which the first motor symptoms developed. Motor symptom severity was assessed using the Hoehn and Yahr scale and the MDS Unified Parkinson’s Disease Rating Scale part III (MDS-UPDRS III) in the medication “ON” state. Cognitive function was evaluated using the Mini-Mental State Examination (MMSE) and the Japanese version of the Montreal Cognitive Assessment (MoCA-J). The presence of RBD was determined with a single-question screening for RBD [11] and the Japanese version of the REM sleep behavior disorder screening questionnaire (RBDSQ-J) [12]. Patients’ subjective experiences of motor symptoms and NMS were assessed using a structured questionnaire, which is described in detail below.

This study was performed in accordance with the Declaration of Helsinki. This human study was approved by Sendai Nishitaga hospital – approval: R2-6. All adult participants provided written informed consent to participate in this study.

### 2.2 Assessment of motor and nonmotor symptoms

We developed a self-administered structured questionnaire to assess the prevalence and onset timing of motor symptoms and NMS in patients with PD. The questionnaire included items addressing cognitive dysfunction as well as symptoms derived primarily from the prodromal PD diagnostic criteria [13,14]. Specifically, four motor symptoms were assessed in the questionnaire, including bradykinesia, balance problems, and tremors, and 34 NMS, such as olfactory dysfunction, memory impairment, language difficulties, sleep disturbances, autonomic dysfunction, and psychotic symptoms. Each symptom was assessed using a single-question, including single-question screening for RBD (Table 1).

**Table 1:** Prevalence and timing of self-reported motor and nonmotor symptoms.

	Prevalence (n = 121)	Occurring timing (n = 78*)	
	Presence	Early phase	Post-motor phase
1. Subjective forgetfulness	64 %	30 %	70 %
2. Objective forgetfulness	40 %	15 %	85 %
3. Short-term memory loss	27 %	11 %	89 %
4. Time disorientation	30 %	19 %	81 %
5. Misplacing objects	45 %	22 %	78 %
6. Delusions of theft	4 %	0 %	100 %
7. Word-finding difficulty	69 %	22 %	78 %
8. Word recall difficulty	67 %	27 %	73 %
9. Trouble with daily tasks	52 %	23 %	77 %
10. Forgetfulness affecting daily life	26 %	14 %	86 %
11. Getting lost in familiar places	5 %	50 %	50 %
12. Improper clothing choices	7 %	25 %	75 %
13. Decline in self-care	33 %	23 %	77 %
14. Irritability	25 %	20 %	80 %
15. Impulsive behavior	16 %	10 %	90 %
16. Stereotypical behavior	25 %	26 %	74 %
17. Changed food preferences	18 %	17 %	83 %
18. Loss of empathy	17 %	38 %	62 %
19. Anxiety	54 %	25 %	75 %
20. Depression	51 %	30 %	70 %
21. Apathy	57 %	29 %	71 %
22. Cognitive fluctuations	41 %	13 %	87 %
23. Presence/ passage hallucination	40 %	11 %	89 %
24. Visual/ auditory hallucinations	36 %	13 %	87 %
25. REM sleep behavior disorder **	57 %	49 %	51 %
26. Sleep disturbances	55 %	44 %	56 %
27. Excessive daytime sleepiness	36 %	30 %	70 %
28. Frequent or urgent urination	61 %	30 %	70 %
29. Urinary incontinence	34 %	24 %	76 %
30. Constipation **	78 %	60 %	40 %
31. Dizziness/ lightheadedness	47 %	29 %	71 %
32. Olfactory dysfunction **	49 %	62 %	38 %
33. Double vision	44 %	38 %	62 %
34. Bradykinesia	94 %	31 %	69 %
35. Repeated falls	60 %	24 %	76 %
36. Tremors	74 %	39 %	61 %
37. Choking on food/ liquids	39 %	28 %	72 %
38. Peripheral edema	39 %	30 %	70 %

* Assessment of the timing of occurrence of each motor and nonmotor symptoms in 78 patients with PD and a disease duration of less than 10 years. The early phase included the pre-motor phase and the phase simultaneous with motor symptoms onset.

** Nonmotor symptoms that were more frequently reported in the early phase than in the post-motor phase.

For each symptom, patients were asked to indicate the time of its onset in relation to the time the questionnaire was taken by selecting from one of the following: <1 year, 1–2 years, 3–5 years, 5–7 years, 7–10 years, or >10 years before the evaluation. These time windows were selected based on a questionnaire used in a previous study by Schrag et al. [5]. We evaluated both the prevalence and the onset times of NMS in PD. To minimize recall bias, questionnaires from participants with MMSE scores <21 and disease durations of >10 years were excluded from the analysis, resulting in 78 of 121 PD patients analyzed.

### 2.3 Cluster analysis

To identify temporal patterns of motor and nonmotor symptom emergence, we conducted a hierarchical cluster analysis using the Ward’s linkage method. Classification variables included age at PD onset and the timing of self-reported NMS onset obtained from questionnaires. PD onset was defined as the first parkinsonian motor symptoms documented in clinical records and the age at PD onset was used as a continuous variable and entered in its original scale. For each NMS, onset was categorized as occurring before, concurrent with, or after the onset of motor symptoms, or not present, and these categories were treated as nominal variables. Therefore, we classified patients into subgroups according to the temporal configurations in which NMS emerged relative to motor onset.

The number of clusters was determined by examining the fusion distances in the dendrogram. We identified the point at which the agglomeration coefficient showed a marked increase, indicating that further merging would combine relatively dissimilar groups. Based on this distance-based criterion, the final cluster solution was selected (Supplementary Fig. 1).

In addition, to validate the stability and reliability of the identified clusters, we conducted a multiscale bootstrap resampling analysis with 5000 replications using the *pvclust* package in R version 4.5.2. Approximately Unbiased (AU) p-values were calculated for each cluster in the hierarchical dendrogram. Clusters with AU p-values greater than 90 % considered to show high statistical stability (Supplementary Fig. 2).

### 2.4 Magnetic resonance imaging analysis and group comparison

We additionally conducted an exploratory voxel-based morphometry (VBM) analysis to examine whether the identified clusters were associated with cortical morphological alterations. VBM was performed in 58 of the 78 patients who underwent MRI within three months of the questionnaire assessment. These 58 patients belonged to the PD subgroups identified in the cluster analysis. The methods used for VBM analysis was described previously [15]. In brief, we acquired three-dimensional T1-weighted images using a 1.5-T MRI scanner (Siemens MAGNETOM Aera). We performed VBM analysis using Statistical Parametric Mapping software (SPM12) implemented in MATLAB R2015a to calculate total brain volume and to compare cortical volume among patient subgroups by a two-sample *t*-test, with age, sex and total intracranial volumes as covariates (uncorrected p < 0.001).

### 2.5 Statistical analysis

The clinical and demographic data were summarized using descriptive statistics. Comparisons of the identified clusters were performed using one-way analyses of variance with *post-hoc* Tukey–Kramer honestly significant difference tests or Kruskal–Wallis tests with *post-hoc* Dunn’s tests with Bonferroni corrections. *P-*values < 0.05 were considered statistically significant. Statistical tests were conducted using JMP Pro 17 (SAS Institute, Cary, NC, USA).

### 2.6 Data sharing

The data that support the findings of this study are not publicly available due to information that could compromise the privacy of research participants but are available from the corresponding author (A.T.) upon reasonable request.

## 3 Results

### 3.1 Clinical and demographic data

A total of 121 patients with PD who had MMSE score of 22 or higher were included in our cohort. This group consisted of 46 males and 75 females, with a mean age of 71.2 (±7.9) and the mean disease duration of 9.1 (±6.1) years. Among them, 78 of the patients had a disease duration of <10 years. These were included in our cluster analysis. The results of this analysis are presented in Table 2.

**Table 2:** Patient characteristics and demographics.

						*Post hoc test p-value*		
	Cluster 1 n = 27	Cluster 2 n = 17	Cluster 3 n = 34	p-value	η^2^	1_2	1_3	2_3
Male sex, n (%)	9 (33)	7 (41)	16 (47)					
Age, years	75.3 ± 4.3	63.8 ± 6.4	74.5 ± 6.1	**<0.0001**	0.4681	**<0.0001**	0.8594	**<0.0001**
Age at onset, years	71.6 ± 4.1	58.1 ± 7.1	68.2 ± 6.7	**<0.0001**	0.4904	**<0.0001**	**0.0823**	**<0.0001**
Disease duration, years	3.7 ± 2.5	5.7 ± 2.7	6.3 ± 2.1	**0.0005**^a^	0.195	**0.0334**	**0.0004**	1.0000
LEDD, mg	525.0 ± 329.3	563.5 ± 336.3	677.5 ± 363.8	0.3128^a^	0.0473	1.0000	0.479	0.858
Hoehn and Yahr scale	2.9 ± 1.1	2.6 ± 0.8	2.8 ± 1.0	0.7157^a^	0.0267	1.0000	1.0000	1.0000
MDS-UPDRS PART Ⅲ (n = 26/ 15/ 25)	25.3 ± 18.8	26.2 ± 20.4	23.1 ± 16.1	0.9346^a^	0.0025	1.0000	1.0000	0.9386
Number of motor and nonmotor symptoms (questionnaire)	12.1 ± 4.8	8.8 ± 5.6	20.4 ± 4.6	**<0.0001**	0.4858	0.0739	**<0.0001**	**<0.0001**
RBDSQ	3.4 ± 2.3	2.9 ± 2.0	5.1 ± 3.1	**0.0190**^a^	0.0859	1.0000	0.1023	**0.0358**
MMSE	26.1 ± 3.2	27.8 ± 2.6	25.9 ± 3.1	0.0906^a^	0.0775	0.2367	1.0000	0.1017
MoCA-J	21.2 ± 4.9	24.1 ± 4.6	21 ± 4.0	**0.0304**^a^	0.0919	0.0714	1.0000	**0.0329**
VBM (n = 20/13/25)								
Total brain volume, ml	944.7 ± 80.2	1048.9 ± 139.8	1004.7 ± 99	**0.02**^a^		**0.029**	0.092	1.0000

Data are presented in mean (standard deviation), unless otherwise specified. Comparison among the cluster groups was calculated by one-way analysis of variance or Kruskal-Wallis test with the post hoc test corrected for Tukey-Kramer honestly significant difference test. A p-value of less than 0.05 was considered statistically significant.

Abbreviations: LEDD, levodopa equivalent daily dose; NMS, nonmotor symptom; RBDSQ, REM sleep behavior disorder screening questionnaire; MMSE, Mini-Mental State Examination; MoCA-J, Japanese version of Montreal Cognitive Assessment; VBM, voxel-based morphometry.

a Nonparametric Kruskal-Wallis test, *post hoc* Dunn's test with Bonferroni correction.

### 3.2 Prevalence and timing of patients’ self-reported motor and nonmotor symptoms

The prevalence (%) of the 38 assessed symptoms in the cohort of 121 PD patients is presented in Table 1 for each symptom. Among the NMS, constipation was the most frequently reported (78 %), followed by word-finding difficulties and subjective cognitive decline (69 % and 65 %, respectively).

When we focused on the 78 PD patients with a disease duration of less than 10 years, so-called prodromal NMS, such as olfactory dysfunction, constipation, and RBD, were the most frequently experienced symptoms that occurred either in the prodromal period or concurrently with the onset of motor symptoms (early phase) (Table 1). In contrast, other NMS were more commonly observed after the development of motor symptoms (postmotor phase). To examine the consistency between self-reported motor symptom onset and clinically documented PD onset, we compared the duration of motor symptoms derived from the questionnaire with the disease duration recorded in medical charts. Overall, the agreement between the two measures was modest (Spearman’s ρ = 0.3954, *p* = 0.0004). The concordance was higher in patients with shorter disease duration (Spearman’s ρ = 0.5132, *p* = 0.0007), but it declined as disease duration increased. This pattern suggests that patients with more advanced disease may have reported the timing of worsening of motor symptoms rather than the emergence of first parkinsonian signs. Notably, we used only clinically documented motor onset in the cluster analysis to ensure consistency and reduce recall bias.

### 3.3 Cluster analysis

**Fig. 1:**
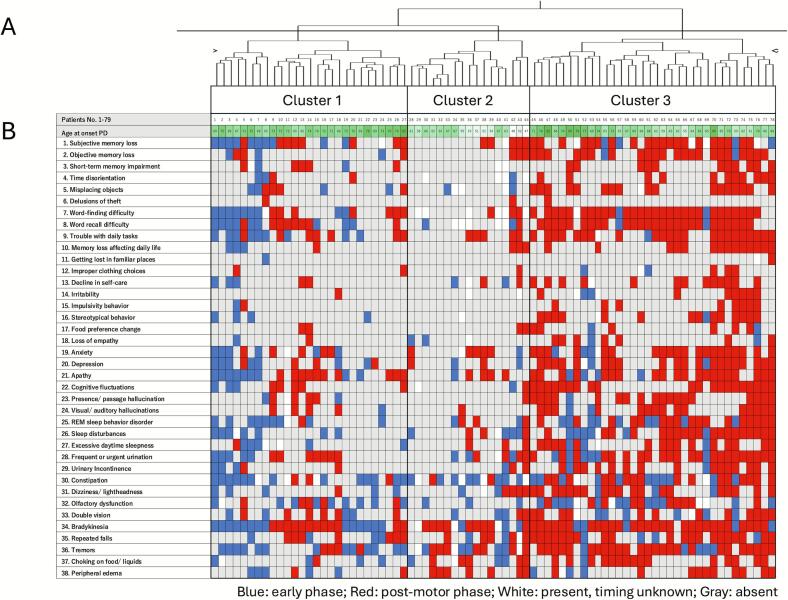
Hierarchical cluster solution and timing of self-reported motor and nonmotor symptoms. (A) Dendrogram of the hierarchical cluster analysis including all 78 patients. Each leaf represents an individual patient. solution. The upper color bar indicates age at onset, color-coded from white (younger) to green (older). The three major branches correspond to Clusters 1, 2, and 3. (B) Heatmap showing the timing of self-reported motor and nonmotor symptoms relative to motor symptom onset. Blue indicates symptoms occurring in the early phase (premotor or concurrent with motor symptom onset), red indicates symptoms occurring in the postmotor phase, white indicates symptoms that are present but for which the onset timing is unknown, and gray indicates absence of the symptom. Black frames indicate patient groups corresponding to the three clusters shown in panel A. (For interpretation of the references to colour in this figure legend, the reader is referred to the web version of this article.)

Cluster analysis identified three temporal patterns of motor and nonmotor symptom emergence (Fig. 1 and Table 2, supplementary Fig. 1). The severity of the motor symptoms was similar between the three clusters. Cluster 1 was the group with the oldest onset age and shortest disease duration. In this cluster, sleep disturbances often preceded motor symptoms, and mood and cognitive dysfunction were found both before and after the onset of motor symptoms. This cluster showed moderate cognitive dysfunction on the MMSE and the MoCA-J and cortical atrophy despite the shorter disease duration. Cluster 2 was the youngest group, with the earliest onset age. In this cluster, predominant NMS preceding motor symptoms was constipation, with occasional olfactory disturbances and RBD. They exhibited the slowest disease progression and their cognitive function was generally better preserved than that of the patients in the other two groups. Cluster 3 was another older-onset group. They suffered the highest average number of NMS, which appeared mainly in the postmotor phase. In particular, there were multiple cognitive dysfunctions, psychiatric symptoms, autonomic dysfunctions, and sleep disturbances. These symptoms became prominent rapidly after the onset of motor symptoms. This group exhibited the highest RBDSQ scores, the highest prevalence of postmotor RBD, and poor cognitive function. In addition, we assessed the internal stability of this clustering solution using bootstrap resampling. While the exact cluster assignments varied across bootstrap iterations, core groups corresponding to each of the three patterns were consistently detected, indicating that the identified symptom-emergence patterns represent statistically supported, reproducible structures rather than an arbitrary partition (supplementary Fig. 2).

### 3.4 Comparison of brain gray matter atrophy between the clusters

**Fig. 2:**
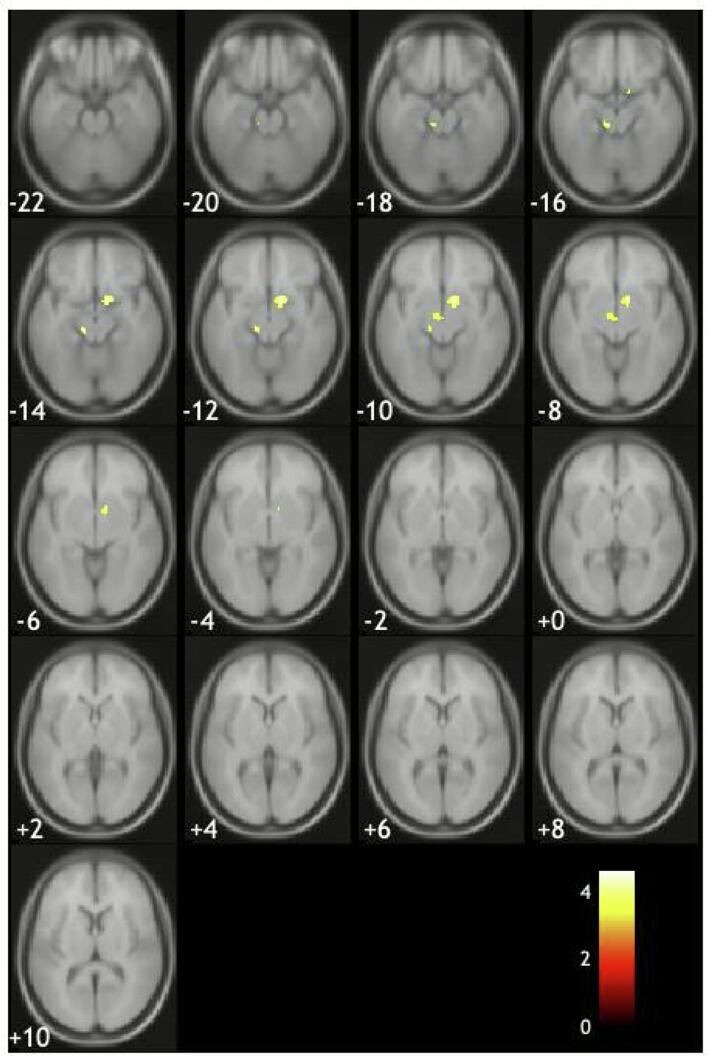
Areas of brain atrophy in Parkinson’s disease patients in Cluster 1 compared with the those in Cluster 2. Basal forebrain and the brainstem regions showed a trend toward atrophy (uncorrected < 0.001).

The number of patients in Cluster 1 was 20, Cluster 2 was 12, and Cluster 3 was 25. Compared with Cluster 2, Cluster 1 showed significantly decreased total brain volume (Table. 2). Voxel based comparison revealed that Cluster 1 had a trend toward atrophy in the basal forebrain and brainstem compared to Cluster 2 (Fig. 2), but these findings did not remain significant after correction for multiple comparisons (FWE *p* < 0.05). No significant differences in gray matter volume were observed between Cluster 2 and Cluster 3.

## 4 Discussion

In this study, we identified three temporal patterns in the the emergence of NMS in PD. Cluster 1 comprised an older-onset group who frequently exhibited sleep problems in the prodromal phase. Cluster 2 was a younger-onset group whose prodromal NMS were mainly constipation, and had benign disease course. Cluster 3 was another older-onset group characterized by the emergence of multiple NMS within a short period after motor symptom onset. There was a difference in the timing of NMS onset in clusters 1 and 3, which preceded or followed the onset of motor symptoms, respectively. However, these two clusters shared clinical characteristics such as older onset age, poor cognitive function, and a high rate of NMS. In contrast, patients in cluster 2 were characterized by younger-onset age, preserved cognitive function, and slower progression of NMS.

Previous research has found that the prodromal phase of PD is characterized by several NMS, including constipation, olfactory dysfunction, and RBD [2]. However, recent studies have shown that so-called prodromal NMS are often absent in PD at the time of diagnosis [5], and the longitudinal patterns of NMS development are highly heterogeneous [6,16]. In this study, cluster 2, characterized by a younger age at onset, exhibited the so-called prodromal NMS predominantly in the early phase. In comparison, the two clusters with an older age at onset were characterized by a greater number of both the so-called prodromal and late NMS, which were observed either before or after the onset of motor symptom. Notably, even the so-called prodromal NMS were frequently observed to develop in the postmotor phase in cluster 3. The finding that older-onset PD patients in cluster 3 develop multiple NMS after the onset of motor symptoms may have important clinical implications. NMS serve as supporting evidence for PD diagnosis. However, many of the older-onset PD patients in cluster 3 did not exhibit NMS at motor symptom onset, which may make early diagnosis more challenging. Recognizing that NMS tend to emerge after the onset of motor symptoms in older patients may help reduce late diagnosis or misdiagnosis in clinical practice.

Pathological studies have shown that onset age significantly affects the distribution of Lewy body (LB) pathology in PD [17]. Braak et al. reported that LB pathology begins in the lower brainstem and the olfactory bulb during the prodromal phase of PD. It then spreads in a bottom-up fashion from the lower brainstem to the upper brainstem and cortex [18]. Subsequently, Halliday et al. identified three patterns of LB progression in PD. They found that age at PD onset is a key factor in determining the pattern of pathological progression. They found that the brainstem-dominant type, which shares similarities with the type reported by Braak et al. [18], is more common in young-onset PD, whereas limbic and cortical types are more frequent among older-onset PD patients [19]. A recent MRI study demonstrated three patterns of cortical atrophy in patients with PD concordant with the typology of Halliday et al. [19,20]. In the present study, cluster 2, the youngest group, was primarily characterized by motor symptoms, with fewer NMS than the other clusters. Clusters 1 and 3 were characterized by multiple NMS and cognitive decline. These results suggest that cluster 2 may predominantly suffer from dopaminergic denervation with minimal extranigral pathology. In contrast, the widespread clinical progression in clusters 1 and 3 is likely associated with similarly widespread pathological progression in the brain, involving the limbic system and neocortex. Therefore, the age of PD onset may be an indicator of the extent of the pathological distribution involved.

Our results raise questions about recent hypotheses regarding the order of onset of motor symptoms and NMS in PD. Recently, Horsager et al. proposed that the sequence of RBD and PD onset is key to distinguishing between two PD subtypes with different patterns of pathological progression [8]. According to their hypothesis, PD patients who exhibit RBD before the onset of PD have gut-to-brain LB pathology that ascends in a symmetrical manner. This PD type is prone to faster disease progression. In contrast, PD patients who exhibit RBD after the development of PD have asymmetrical onset of brain LB pathology that descends in a top–down manner to the gut. This group shows slower disease progression (the brain-first and body-first (BVB) PD hypothesis) [21]. In our study, cluster 1 showed premotor RBD and poor cognitive function; in the majority with available MRI, this cluster also tended to exhibit greater cortical atrophy, findings seemed consistent with the BVB hypothesis. However, another older onset malignant group, cluster 3, had post motor RBD, and this result is contrary to the BVB hypothesis. While it is clear that PD without RBD represents a milder form of the disease [22], it remains unclear whether postmotor RBD serves as a marker of a good or poor prognosis in PD [[23], [24], [25]]. Recent studies have reported that PD patients with postmotor RBD may have a more severe clinical course than those with premotor RBD [26,27]. Given the diversity of PD, it seems that classifying PD patients solely by the order of RBD and motor symptoms may be an oversimplification [28].

There were some limitations to this study. First, although bootstrap resampling supported the presence of three core symptom-emergence patterns, the sample size for the cluster analysis was relatively small, which may limit the stability and generalizability of the identified subgroups. Second, the data were retrospectively self-reported symptoms. Although we excluded PD patients with severe cognitive impairments, recall biases cannot be eliminated from this type of data. Third, although we created our structured questionnaire based on the current prodromal PD research criteria [13,14], it has not undergone formal longitudinal validation, and further validation of the questionnaire is needed. Fourth, because the MRI sample size was relatively small, and the VBM analyses were conducted using an uncorrected statistical threshold, neuroimaging findings should be interpreted cautiously. Fifth, we did not perform polysomnography to assess RBD. While the RBDSQ-J has high sensitivity and specificity for RBD detection [11], it remains a less objective and accurate form of diagnosis than polysomnography. Sixth, this study was performed in an inpatient setting, which may have resulted in an overrepresentation of older patients. This, combined with the single-center design, will have reduced the generalizability of our findings. These factors may have influenced the observed symptom progression patterns. Finally, the cross-sectional design does not allow direct assessment of longitudinal change, thus the identified patterns should be interpreted as temporal associations rather than true progression trajectories. Future longitudinal studies are needed to prospectively ascertain the onset and sequence of individual symptoms and to determine whether the identified symptom-emergence patterns predict prognosis in PD.

In conclusion, we observed three temporal occurrence patterns of motor symptoms and NMS in PD. A younger-onset PD group showed a typical prodromal to late-stage NMS emergence pattern, but two older-onset PD groups showed a wide variety of NMS regardless of the onset times of motor symptoms and cognitive dysfunction. Understanding the combination patterns and order of appearance of motor symptoms and NMS will contribute to better classification of PD subtypes and more individualized PD treatment.

## 5 Financial disclosures of all authors for the past year

YS has no financial disclosures to report. TB reports honoraria from Ono Pharmaceutical Co., Ltd., AbbVie Inc., Kyowa Kirin Co., Ltd., Sumitomo Pharma, Takeda Pharma, Otsuka Pharma, Eisai Co., Ltd., outside the submitted work. IK has no financial disclosures to report. TT reports honoraria from Kyowa Kirin Co., Ltd. and Nihon Medi-physics Co., Ltd., outside the submitted work. HO has no financial disclosures to report. TH reports grants from Biogen, honoraria from Takeda Pharma, Sumitomo Pharma, Kyowa Kirin Co., Ltd., Eisai Co., Ltd., Kowa Co., Ltd., outside the submitted work. KS has no financial disclosures to report. AT reports grants from Kyowa Kirin Co., honoraria from AbbVie Inc., Eisai Co., Ltd., Kyowa Kirin Co., Ltd., Ono Pharmaceutical Co., Ltd., Sumitomo Pharma, Takeda Pharma, outside the submitted work.

## 6 Data Availability Statement

The data that support the findings of this study are not publicly available due to information that could compromise the privacy of research participants but are available from the corresponding author (A.T.) upon reasonable request.

## 7 Funding agencies

This work was supported by Grants-in-Aid from the Research Committee of CNS Degenerative Diseases; Research on Policy Planning and Evaluation for Rare and Intractable Diseases; Health, Labor, and Welfare Sciences Research grants; and the Ministry of Health Labor and Welfare, Japan.

## CRediT authorship contribution statement

**Yoko Sugimura:** Formal analysis, Data curation, Methodology, Resources, Visualization, Writing – original draft. **Toru Baba:** Conceptualization, Data curation, Methodology, Resources, Visualization, Writing – review & editing. **Tomoko Totsune:** Data curation, Resources, Writing – review & editing. **Hideki Oizumi:** Resources, Writing – review & editing. **Takafumi Hasegawa:** Supervision, Writing – review & editing. **Kyoko Suzuki:** Supervision, Writing – review & editing. **Atsushi Takeda:** Supervision, Resources, Funding acquisition, Writing – review & editing.

## Declaration of competing interest

The authors declare the following financial interests/personal relationships which may be considered as potential competing interests: Toru Baba reports a relationship with Ono Pharmaceutical Co Ltd that includes: speaking and lecture fees. Toru Baba reports a relationship with AbbVie Inc that includes: speaking and lecture fees. Toru Baba reports a relationship with Kyowa Kirin Co Ltd that includes: speaking and lecture fees. Toru Baba reports a relationship with Sumitomo Pharma Co Ltd that includes: speaking and lecture fees. Toru Baba reports a relationship with Takeda Pharmaceutical Company Limited that includes: speaking and lecture fees. Toru Baba reports a relationship with Otsuka Pharmaceutical Co Ltd that includes: speaking and lecture fees. Toru Baba reports a relationship with Eisai Co Ltd that includes: speaking and lecture fees. Tomoko Totsune reports a relationship with Kyowa Kirin Co Ltd that includes: speaking and lecture fees. Tomoko Totsune reports a relationship with Nihon Medi-Physics Co Ltd that includes: speaking and lecture fees. Takafumi Hasegawa reports a relationship with Biogen that includes: funding grants. Takafumi Hasegawa reports a relationship with Takeda Pharmaceutical Company Limited that includes: speaking and lecture fees. Takafumi Hasegawa reports a relationship with Sumitomo Pharma Co Ltd that includes: speaking and lecture fees. Takafumi Hasegawa reports a relationship with Kyowa Kirin Co Ltd that includes: speaking and lecture fees. Takafumi Hasegawa reports a relationship with Eisai Co Ltd that includes: speaking and lecture fees. Takafumi Hasegawa reports a relationship with Kowa Co Ltd that includes: speaking and lecture fees. Atsushi Takeda reports a relationship with Kyowa Kirin Co Ltd that includes: funding grants. Atsushi Takeda reports a relationship with AbbVie Inc that includes: speaking and lecture fees. Atsushi Takeda reports a relationship with Eisai Co Ltd that includes: speaking and lecture fees. Atsushi Takeda reports a relationship with Kyowa Kirin Co Ltd that includes: speaking and lecture fees. Atsushi Takeda reports a relationship with Ono Pharmaceutical Co Ltd that includes: speaking and lecture fees. Atsushi Takeda reports a relationship with Sumitomo Pharma Co Ltd that includes: speaking and lecture fees. Atsushi Takeda reports a relationship with Takeda Pharmaceutical Company Limited that includes: speaking and lecture fees. If there are other authors, they declare that they have no known competing financial interests or personal relationships that could have appeared to influence the work reported in this paper.
