# Supplementary material for: Temporal patterns of motor and nonmotor symptom emergence in Parkinson’s disease: a cluster analysis
Source: Clin Park Relat Disord. 2026 Jan 6;14:100423. doi: 10.1016/j.prdoa.2026.100423 (PMC12830228; doi:10.1016/j.prdoa.2026.100423)
Supplement: Supplementary Data 1 [file mmc1.docx]

**Supplementary figure 1: Agglomeration coefficient plot for determining the optimal number of clusters**

The marked increase in the agglomeration coefficient at the final merging step (from 3 to 2 clusters; dashed line) indicates that further merging would combine relatively dissimilar groups, supporting the choice of a three-cluster solution in the main analysis.


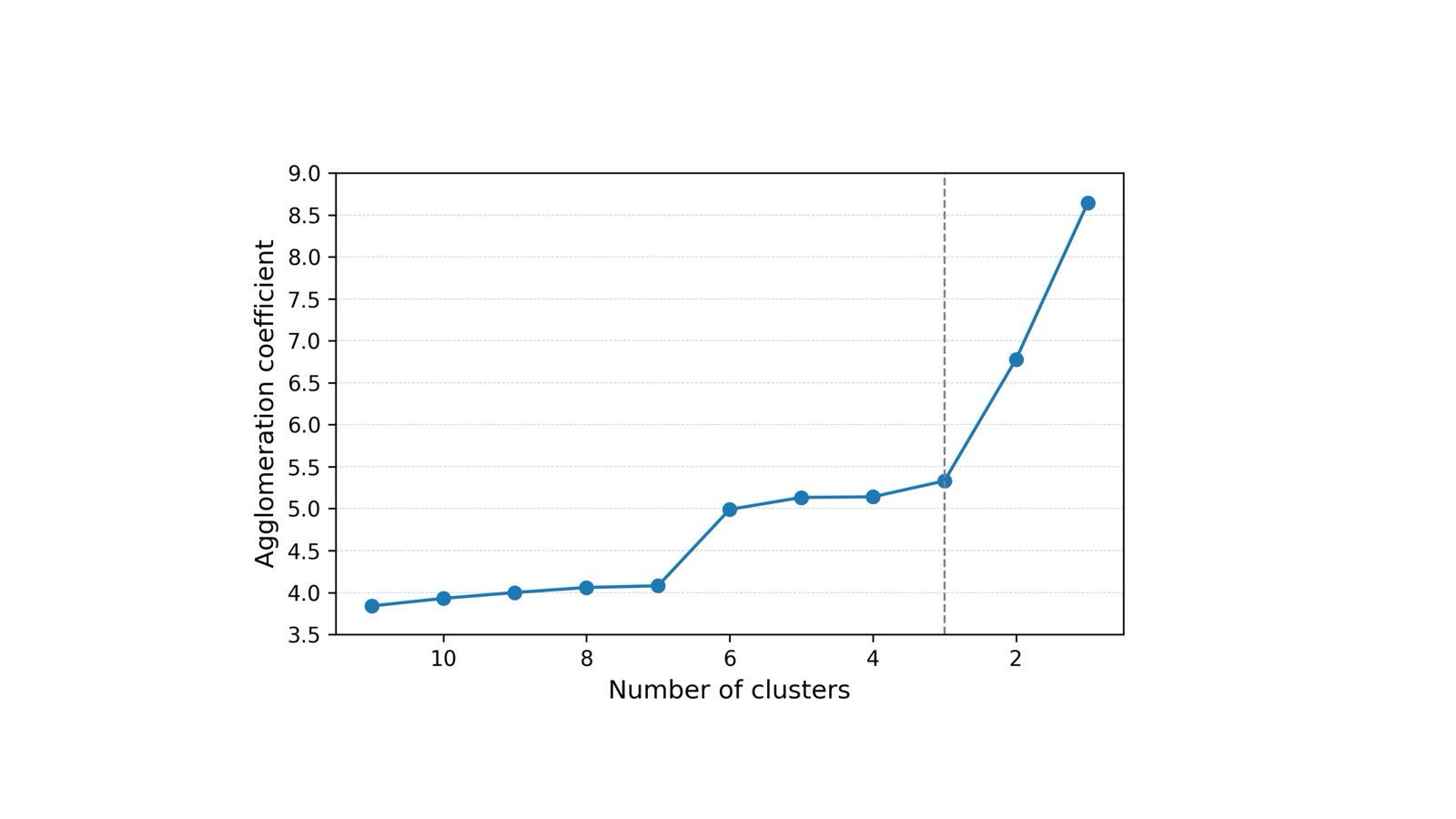


**Supplementary figure 2: Multiscale Bootstrap Validation of the Hierarchical Clustering Solution**

Multiscale bootstrap resampling was used to assess the stability of the hierarchical clustering solution. In the dendrogram, clusters with Approximately Unbiased (AU) p-values ≥ 90% are highlighted by red rectangles, indicating statistically stable clusters. The corresponding subjects are indicated by black rectangles in the heatmap below. The heatmap displays the timing of endorsed motor and nonmotor symptoms relative to motor symptom onset (blue = premotor or concurrent with motor symptoms; red = post-motor; white = unknown onset; gray = absence of the symptom).
